# Confirmation that “*Brachyspira hampsonii*” clade I (Canadian strain 30599) causes mucohemorrhagic diarrhea and colitis in experimentally infected pigs

**DOI:** 10.1186/1746-6148-10-129

**Published:** 2014-06-10

**Authors:** Matheus O Costa, Janet E Hill, Champika Fernando, Hollie D Lemieux, Susan E Detmer, Joseph E Rubin, John C S Harding

**Affiliations:** 1 Department of Veterinary Microbiology, University of Saskatchewan, 52 Campus Dr., S7N 5B4 Saskatoon, SK, Canada; 2 Department of Large Animal Clinical Sciences, University of Saskatchewan, Saskatoon, SK, Canada; 3 Department of Veterinary Pathology, University of Saskatchewan, Saskatoon, SK, Canada

**Keywords:** “*Brachyspira hampsonii”*, Swine dysentery, Mucohemorrhagic, Bloody, Diarrhea, Colitis, Pig, Porcine

## Abstract

**Background:**

“*Brachyspira hampsonii*”, discovered in North America in 2010 associated with dysentery-like illness, is an economically relevant swine pathogen resulting in decreased feed efficiency and increased morbidity, mortality and medication usage. “*B. hampsonii”* clade II strain 30446 has been shown to be causally associated with mucohemorrhagic diarrhea and colitis. Our objectives were to determine if “*Brachyspira hampsonii*” clade I strain 30599 is pathogenic to pigs, and to evaluate the relative diagnostic performance of three *ante mortem* sampling methodologies (direct PCR on feces, PCR on rectal GenoTube Livestock swabs, *Brachyspira* culture from rectal swabs). Five-week old pigs were intragastrically inoculated thrice with 10^8^ genomic equivalents *"B. hampsonii"* (n = 12), or served as sham controls (n = 6). Feces were sampled and consistency assessed daily. Necropsies were performed 24 h after peak clinical signs.

**Results:**

One pig died due to unrelated illness. Nine of 11 inoculated pigs, but no controls, developed mucoid or mucohemorrhagic diarrhea (MHD). Characteristic lesions of swine dysentery were observed in large intestine. *“B. hampsonii”* strain 30599 DNA was detected by qPCR in feces of all inoculated pigs for up to 6 days prior to the onset of MHD. The organism was isolated from the feces and colons of pigs demonstrating MHD, but not from controls. *B. intermedia* was isolated from inoculated pigs without MHD, and from 5 of 6 controls.

**Conclusions:**

We conclude that “*Brachyspira hampsonii*” clade I strain 30599 is pathogenic and causes mucohemorrhagic diarrhea and colitis in susceptible pigs. Moreover, the three sampling methodologies performed similarly. GenoTube Livestock, a forensic swab designed to preserve DNA during shipping is a useful tool especially in settings where timely transport of diagnostic samples is challenging.

## Background

The first published description of swine dysentery (SD) appeared in 1921, although the causative agent was unknown at the time [1]. In 1971, *Treponema hyodysenteriae* (later renamed *Brachyspira hyodysenteriae*) was identified as the pathogen responsible for the syndrome [2,3]. Until the early 1990′s, SD was considered a major production-limiting disease in North American commercial swine farms. Since the mid-2000's, swine producers and veterinarians have observed the re-emergence of mucohemorrhagic diarrhea (MHD) and colitis in commercial farms [4-7]. North American diagnostic laboratories observed an increase in the number of cases of *Brachyspira hyodysenteriae* diarrhea submitted, as well as cases associated with “atypical” *Brachyspira* spp. [8]. Further characterization of these atypical isolates led to the description of a novel *Brachyspira* species, provisionally named “*Brachyspira hampsonii”.* It has been proposed that this new species is comprised of two different phylogenetic clades, sharing 96% sequence identity in the NADH oxidase (*nox)* gene sequence [9].

Mucohemorrhagic diarrhea and colitis, indistinguishable from SD has been experimentally reproduced in pigs with “*B. hampsonii”* clade II isolates from Canada and the United States [10,11]*.* Burrough *et al.* has also reproduced SD experimentally in mice [12] and in pigs [11] using strongly β-hemolytic strains initially identified as *B. intermedia* based on PCR [13], which have since been confirmed to be “*B. hampsonii”* clade I [14]. These data suggests that clade I is pathogenic to pigs, but further studies are required to confirm this, evaluate additional strains and to characterize the disease including patterns of shedding.

The first Canadian diagnosis of *“B. hampsonii”* clade I was in November 2011 from grow-finish pigs with bloody, mucoid diarrhea. This was a unique event, since all previous *“B. hampsonii”* cases diagnosed by our laboratory were of clade II. The isolate recovered from this diagnostic case was designated “30599”. Since its first diagnosis in Canada, *“B. hampsonii”* clade I (strain 30599) has been identified, isolated or both, in the absence of other *Brachyspira* spp., in feces or tissues from 39 cases of diarrhea from 15 farms in western Canada. Given the distance between farms and diagnostic laboratories in western Canada, this may be an underestimate of the number of farms affected by the organism. One of the main obstacles to obtaining high quality diagnostic samples and reliable results is the transit time required to ship samples to a diagnostic laboratory [15,16]. For PCR based diagnostics, sample quality may be improved by immediate preservation of target DNA in the sample and controlling the growth of opportunistic organisms. A forensic swab (GenoTube Livestock; Prionics, Switzerland) capable of rapidly drying the sample and providing minimal DNA loss after long-term storage without refrigeration may be a potential tool for aiding the diagnosis of infectious diseases by PCR in biologic samples shipped long distances to diagnostic laboratories.

The objectives of this study were to determine the pathogenicity of “*Brachyspira hampsonii*” clade I (strain 30599) when inoculated in naïve pigs, and to investigate the performance of GenoTube Livestock swabs for *ante mortem* sampling of pigs for PCR-based detection of *“Brachyspira hampsonii*” clade I. In order to accomplish these primary objectives, we also developed and validated a PCR assay for the detection and quantification of “*Brachyspira hampsonii*” clade I (strain 30599).

## Methods

This work was approved by the University of Saskatchewan’s Animal Research Ethics Board and adhered to the Canadian Council on Animal Care guidelines for humane animal use (permit #20110038) and ARRIVE Guidelines (Animal Research: Reporting In Vivo Experiments; Additional file 1).

### Source of “*B. hampsonii*” strain 30599 inoculum

*“B. hampsonii”* clade I strain 30599 was isolated from a pig with mucoid diarrhea in Alberta, Canada in November, 2011. PCR for *B. hyodysenteriae*[13], *B. pilosicoli*[10] and *“B. hampsonii”* clade II [10] performed on case tissues returned negative results. Sections of small and large intestines tested negative for *Salmonella* (culture on brilliant green agar following enrichment with selenite broth), *Lawsonia intracellularis* (PCR) [17], and porcine circovirus type 2 (PCV2, immunohistochemistry) [18] at the Prairie Diagnostic Service Inc. (PDS), Saskatoon, SK. Culture of colon tissue on selective media resulted in areas of strong β-hemolysis, which were sub-cultured to obtain an isolate. Sequencing of the *nox*, *cpn*60 and 16S rRNA genes from this isolate demonstrated that it was similar to, but phylogenetically distinct from *“B. hampsonii”* clade II. Whole genome sequencing of the isolate was performed (NCBI BioProject PRJNA188379) and confirmed its affiliation with what has recently been proposed as clade I of *“B. hampsonii”*[9].

### Experimental inoculation

The experimental inoculation was performed as described previously (“Pure Broth Culture Inoculation” in [10]). Briefly, 18 five-week old pigs (16 male, 2 inadvertently female) were obtained from a commercial farm in Saskatchewan, Canada. No history of MHD or previous diagnosis of *Brachyspira* spp. associated diarrhea was reported, and fecal samples collected from nursery (n = 20) and grow-finish (n = 10) pigs prior to the commencement of the study tested negative for *Brachyspira* spp. by culture and species-specific qPCR for *B. hyodysenteriae, B. pilosicoli* and *“B. hampsonii”* (data not shown). Upon arrival at the Animal Care Unit at the University of Saskatchewan, the pigs were allocated randomly to control (CTRL, n = 6) and inoculated (INOC, n = 12) groups housed in separate rooms with 3 or 2 pigs per pen, respectively. Group sizes were based on results of past experiments and provided an experimental power of 0.83 based on the assumptions that 8/12 INOC and 0/6 CTRL pigs would develop MHD and alpha = 0.05. One female pig was assigned to each group. Pigs were acclimated to their new diet and room environment for 7 days prior to inoculation, and were offered *ad libitum* water and commercially prepared, non-medicated, pelleted starter diet (Whole Earth Pig Starter, Federated Cooperative Ltd., Saskatoon, Canada) formulated with less than 15% soybean meal for the duration of the experiment.

Pigs were sedated with 8 mg/kg azaperone IM (Stresnil, Vetoquinol Canada Inc., Lavaltrie, Quebec) then inoculated by gastric tube on three consecutive days (day (D) 0, 1 and 2). INOC received 10 mL of frozen JBS broth containing 6.10 × 10^8^ (D0), 4.14 × 10^8^ (D1) and 4.24 × 10^8^ (D2) genome equivalents of *“B. hampsonii”* strain 30599, followed by 40 mL of PBS (0.1 M, pH 7). CTRL received 10 ml of sterile JBS broth followed by 40 mL of PBS. For both groups, feed was removed 16 h prior to inoculation to increase gastric motility.

Feces and rectal swabs were collected on day −8, −5, −2 and 0 prior to inoculation, and daily thereafter until the end of the experiment. Fecal consistency was scored daily as: 0 = formed, normal; 1 = soft, wet cement consistency; 2 = runny or watery diarrhea; 3 = mucoid diarrhea; 4 = mucohemorrhagic diarrhea. Daily clinical assessment was performed on all pigs. Rectal temperature, responsiveness to external stimuli (e.g. the presence of researcher in the pen) and skin colour were recorded. Body weight was measured on D0 and D8. INOC pigs were humanely euthanized by cranial captive bolt and exsanguination approximately 24 hours after the development of mucoid or mucohemorrhagic diarrhea. INOC pigs that did not demonstrate MHD were euthanized on D13; CTRL pigs on D14.

### Pathological assessments

A complete necropsy was performed and the gastrointestinal tract was completely removed from stomach to rectum, including mesenteric lymph nodes. Special attention was paid to the cecum and spiral colon which was completely linearized and divided into thirds (proximal, apex and distal). The mucosal surfaces of the large intestines were assessed for the presence of characteristic lesions of SD including hyperemia, congestion, edema, necrosis, fibrin and mucus by a single pathologist (SED). A blinded histologic evaluation of colon and cecum was performed by the same pathologist. Tissue samples were fixed in 10% buffered formalin for 24 hours and paraffin embedded. All microsectioned tissues were stained with Hematoxylin-Eosin (H&E) and a serial section of the spiral colon was also Warthin-Faulkner (WF) silver stained. Lesions in the spiral colon and cecum were scored based on the severity of the inflammation and necrosis: 0 = no lesions; 1 = minimal to mild necrosis of superficial enterocytes with minimal inflammatory infiltrates; 2 = moderate necrosis and attenuation of enterocytes with mild to moderate inflammatory infiltrates; 3 = severe necrosis (erosion or ulceration present) with moderate inflammatory infiltrates predominantly consisting of neutrophils. The presence of *Brachyspira*-like organisms was also scored from 0 to 3 in WF stained sections: 0 = no spirochetes observed; 0.5 = a single gland contained a few spirochetes; 1 = small numbers of spirochetes in multiple glands; 2 = many spirochetes within several glands; 3 = many spirochetes forming thick mats in numerous glands.

### Microbiological assessments

Gastrointestinal tissues collected at termination were screened for the presence of other relevant swine enteric pathogens, including *Lawsonia intracellularis* (PCR, ileum), porcine circovirus type 2 (PCV2, immunohistochemistry, ileum and mesenteric lymph node) and *Salmonella* spp. (culture, ileum). All methods were the same as described above. PRRSv IgG antibody and RNA concentration were measured by ELISA (IDEXX PRRS X3, IDEXX Laboratories Inc., Westbrook, ME) and PCR (Tetracore Inc., Rockville, MD), respectively, at PDS in sera collected on D0 and at termination.

### Quantification of *“B. hampsonii”* clade I strain 30599

Details on the development and validation of the strain-specific qPCR assay are given in the Results. DNA for PCR was extracted from feces and colon tissue using the QIAmp DNA stool mini kit (Qiagen Inc., Toronto, ON), and from cultured bacteria and colon tissue using DNEasy blood and tissue kit (Qiagen Inc.). SYBR green real-time qPCR detection of *“B. hampsonii”* strain 30599 was performed on a Bio-Rad MyiQ thermocycler in reactions containing 1× SYBR Green Supermix (Bio-Rad Laboratories (Canada) Ltd., Mississauga, ON), 400 nM each primer JH0436 (5'-AAA GTG CCA CAG GCA ATG TA-3′) and JH0437 (5′-TGC AAG ATT AGA CGG AGC AA-3′)) and 2 μL of template DNA, in a final volume of 25 μL. All qPCR reactions were run on a plate containing a no-template control and a standard curve composed of target-containing plasmids at concentrations of 10^0^ to 10^7^ copies/reaction. All reactions were performed in duplicate. Thermocycling parameters included an initial denaturation (95°C for 3 min.), followed by 40 cycles of 95°C for 15 sec., 63°C for 15 sec., 72°C for 15 sec., and a final extension at 72°C for 5 min. A dissociation curve was subsequently performed for 81 cycles at 0.5°C increments from 55°C to 95°C. Fluorescent signals were measured every cycle at the end of the annealing step and continuously during the dissociation curve data collection. All resulting data was analyzed using iQ5 Optical System Software (Bio-Rad Laboratories (Canada) Ltd., Mississauga, ON).

### *Brachyspira* culture

Isolation of *Brachyspira* spp. was performed on BJ agar from feces and colonic mucosa, as previously described [10]. Presence of *Brachyspira* growth in zones of β-hemolysis was confirmed by dark-field microscopy, and genus-specific PCR targeting the *nox* gene [19] followed by DNA sequencing using the amplification primers.

### Comparison of *ante mortem* sampling techniques

Three *ante mortem* sampling and detection methods were chosen for comparison: rectal swabs (CultureSwab Liquid Stuart, BD Canada, Mississauga, ON) for culture on selective agar, qPCR on DNA extracted from fecal samples (200 mg samples, QIAmp DNA stool mini kit, Qiagen Inc.), and qPCR on DNA extracted from GenoTube swabs. Samples were collected D-8, D-5, D-2 and D0 prior to inoculation, and every other day thereafter to D12 in INOC pigs. Targeted sampling was employed in order to sample pigs with different fecal scores. On each day, samples were simultaneously collected from pigs with no diarrhea (n = 2), pigs with “wet-cement” or watery diarrhea (n = 2) and pigs with mucoid and mucohemorrhagic diarrhea (n = 2). If one or more category was not observed at the time of collection, pigs with the closest available fecal score on that day were sampled. The pigs from which samples were collected were blocked by pen.

To simulate a field situation where samples would be transported to the diagnostic laboratory over 24 h, culture swabs and feces were refrigerated at 4°C (to mimic shipment with ice packs), while GenoTube swabs were stored at room temperature for 24 h prior to processing. GenoTube swabs were expressed into 1.4 mL lysis buffer (buffer ASL, QIAmp DNA stool mini kit, Qiagen Inc., Toronto, ON) in a 2 mL microcentrifuge tube by pressing the swab head against the inside wall of the tube while twisting. To ensure consistent sample expression, swabs were turned exactly ten times each (5 clockwise and 5 counter clockwise). Following expression, extraction proceeded according to the kit instructions. DNA extracts (2 μL each) from feces and GenoTube swabs were used as a template for *“B. hampsonii”* clade I specific qPCR analysis. Culture swabs were used to inoculate BJ agar plates.

### Statistical analysis

Statistical analysis was performed using SPSS v19.0 (SPSS Inc., Chicago, IL) or Stata v13 (StatCorp, College Station, TX). Group differences in the number of pigs demonstrating MHD (fecal consistency score 4) was compared using a Fisher’s exact test. The same test was used to compare the presence or absence of specific gastrointestinal lesions based on gross and histopathological assessments. Continuous variables including average daily gain (ADG) and the concentration of “*B. hampsonii”* strain 30599 DNA detected by qPCR were non-parametrically distributed and compared between groups using a Kruskal-Wallis Analysis of Variance. Spirochete scores in colon were compared with a Mann–Whitney U test. To compare the severity of gross lesions among proximal, apex and distal colon segments, a Wilcoxon matched-pairs signed-rank test was used after assigning negative, mild, moderate and severe lesion scores as 0, 1, 2, 3 respectively. For all analyses, *P* < 0.05 was considered as statistically significant.

## Results

### Development of *“B. hampsonii”* clade I strain 30599 specific SYBR green PCR assay

A strain-specific, SYBR green quantitative real time PCR assay was developed to quantify ”*B. hampsonii“* strain 30599 DNA. Primers JH0436 (5′-AAA GTG CCA CAG GCA ATG TA-3′) and JH0437 (5′-TGC AAG ATT AGA CGG AGC AA-3′) were designed to target a 176 bp region within a predicted open reading frame in the *“B. hampsonii”* strain 30599 genome encoding a hypothetical protein (Genbank accession WP_008726773). The target region lies within an approximately 15.8 kb region of the genome identified as being unique to strain 30599 in a comparison of the whole genome sequences of clade I strain 30599 (BioProject PRJNA187424) and clade II strain 30446 (BioProject PRJNA169353). The target sequence has no significant sequence identity to *“B. hampsonii”* clade II strain 30446, and is only 90% identical at the nucleotide level to a sequence within the genome of *B. intermedia* PWS/A (ATCC 51140^T^). No other significant sequence identities to other *Brachyspira* species were identified.

An optimal annealing temperature of 63°C was determined, and a linear standard curve was obtained over a range of 10^0^ to 10^7^ target copies per PCR reaction using a ten-fold dilution series of cloned target amplicon in plasmid pGEM T Easy as template.

Analytical specificity was determined initially by applying the primers to genomic DNA from *B. hyodysenteriae* ATCC 27164^T^*, B. pilosicoli* ATCC 51139^T^*, B. intermedia* ATCC 51140^T^*, B. innocens* ATCC 29796^T^*, B. murdochii* ATCC 51284^T^, and *“B. hampsonii”* clade II strain 30446. No amplification was detected from any template other than *“B. hampsonii”* clade I strain 30599. Further validation of the analytical specificity of the *“B. hampsonii”* strain 30599 specific qPCR involved its application to fecal samples from previous clinical cases determined to be positive for *“B. hampsonii“* strain 30599 by amplification and sequencing of the *nox* gene using previously published primers (n = 25) [19], and fecal samples from healthy pigs from the source farm that were confirmed negative for *Brachyspira* by the same method (n = 30). All (30/30) of the negative samples were found negative by the strain specific qPCR, and 24 of 25 of the positive samples were found positive by the strain specific qPCR, demonstrating significant agreement between the methods.

### Acclimation period

*Brachyspira* culture was performed on all pigs on four days during the pre-inoculation acclimation period. *B. intermedia* (97% *nox* gene sequence identity over 810 bp to *B. intermedia* ATCC 51140^T^) was isolated from one INOC and one CTRL pig on D-2, and from one INOC on D0. *”B. pulli*” (98% nucleotide identity over 848 bp of *nox* to strain AN304/04) was isolated from one INOC on D-2 and D0. *“B. pulli*” is the provisional name for a spirochete originally identified in birds [20], which has recently also been isolated from pigs [21]. One CTRL had watery diarrhea on D-8 and D-7, immediately after arrival in the animal care facility, but *Brachyspira* was not isolated, and normal feces were observed during all other days pre-inoculation.

### Clinical observations

One INOC pig presented with tachypnea and dyspnea on D3 and D4, and for welfare reasons was euthanized and necropsied on D5. Necropsy findings indicated aspiration of feed at the tracheal bifurcation. Data from this pig were removed from all analyses.

**Figure 1:**
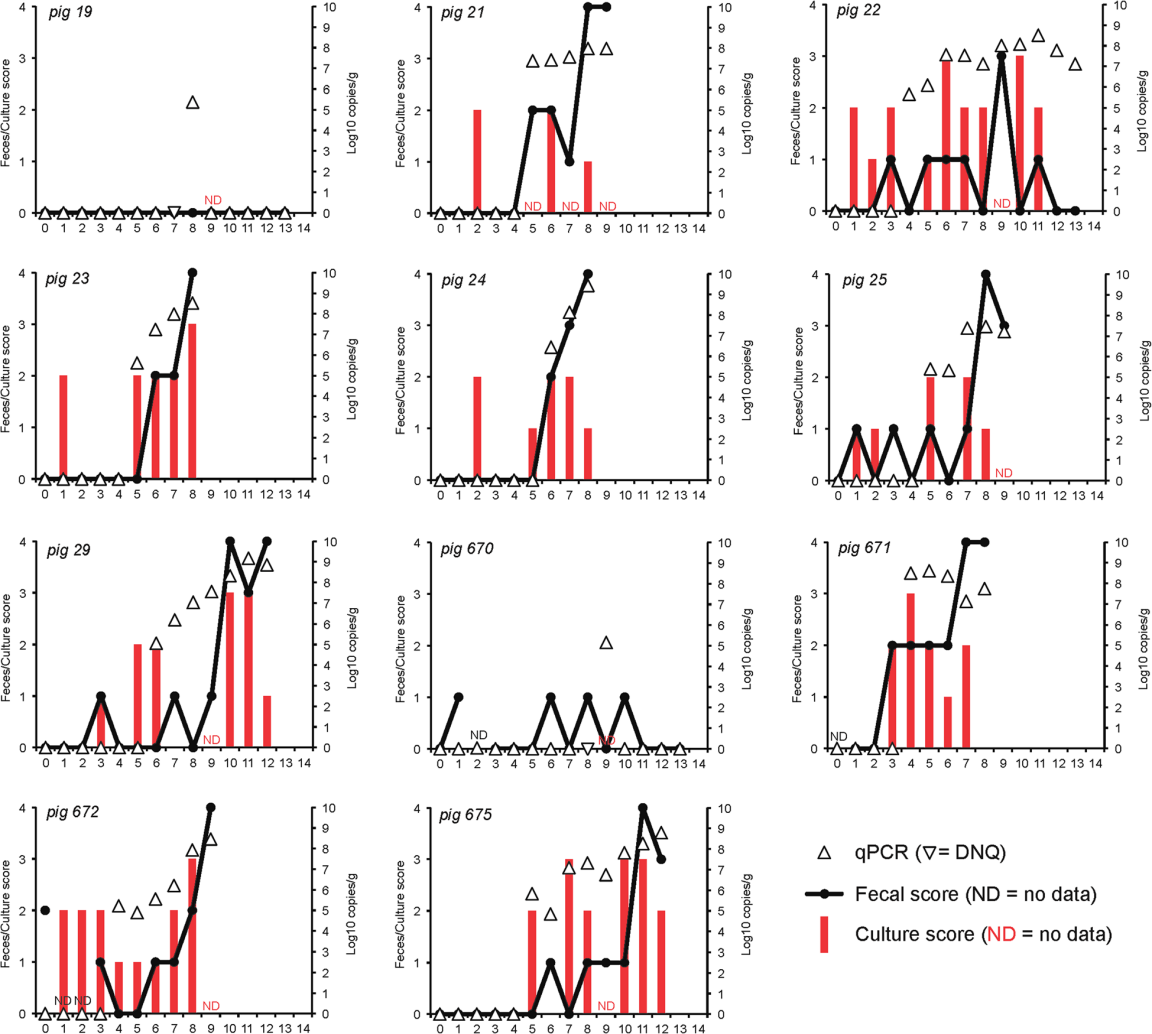
**Fecal consistency scores, culture results and fecal concentration of *“Brachyspira hampsonii” *strain 30599 following inoculation.** Fecal consistency scores (line, left ordinate: 0 = formed, normal; 1 = soft, wet cement consistency; 2 = runny or watery; 3 = mucoid diarrhea; or 4 = bloody diarrhea), semi-quantitative fecal culture score (red bars, left ordinate: 0 = negative; 1 = less than 10 colonies/1° streak; 2 = less than 10 colonies/2° streak; 3 = less than 10 colonies/3°streak; 4 = less than 10 colonies/4° streak), and strain 30599 DNA concentration in feces (triangles, right ordinate; upside down triangles indicate DNQ) are shown. Days post-inoculation are shown on the abscissa. ND = no data. Pig IDs are indicated in the upper left corner of each panel.

**Figure 2:**
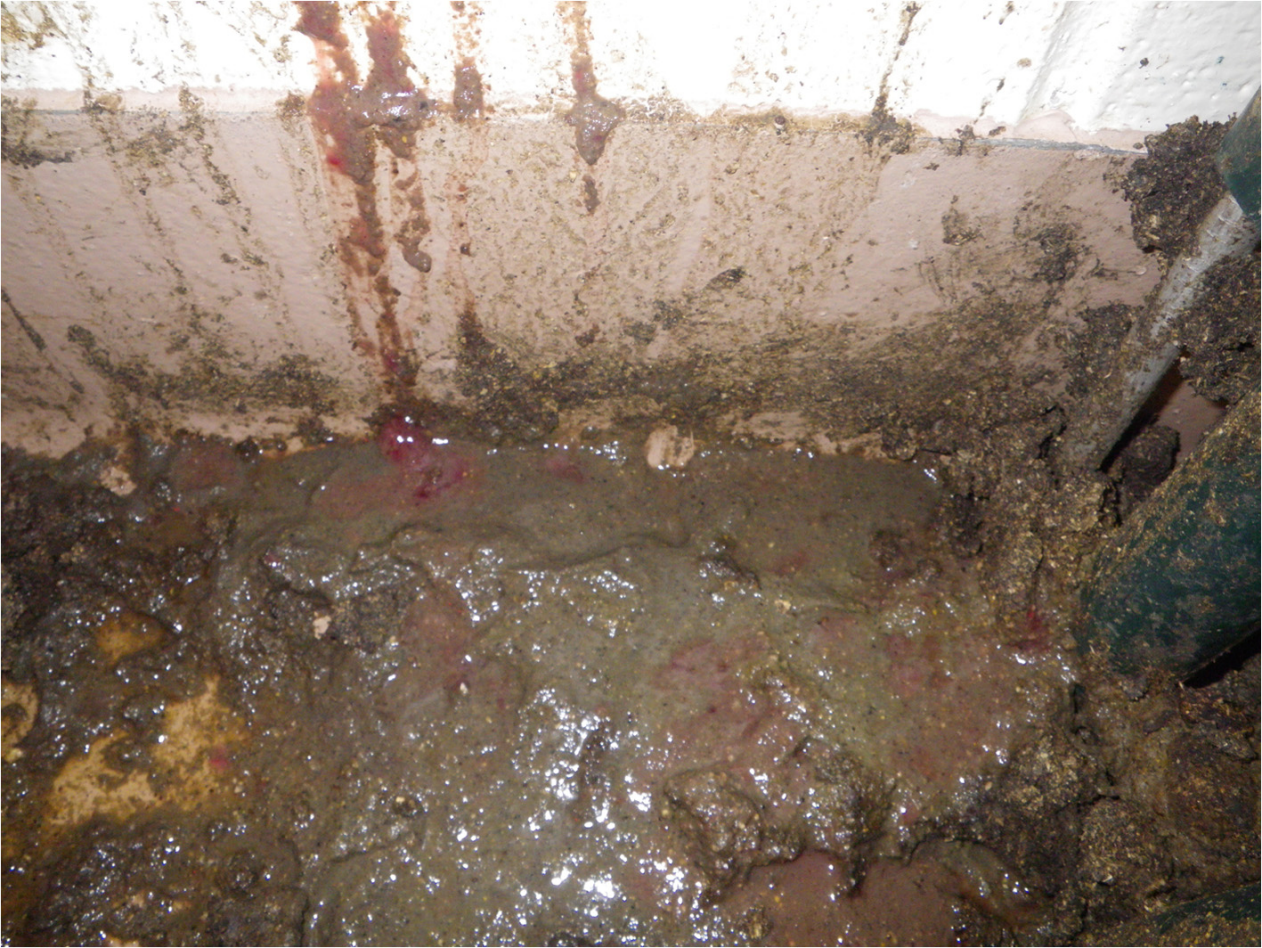
**Mucohemorrhagic diarrhea.** Accumulation of loose and runny feces with mucus and frank blood. Pig #675, 11 days post inoculation, day of first observation of mucohemorrhagic diarrhea, 7th day of *“B. hampsonii”* strain 30599 shedding, feces culture positive (3+ strongly beta-hemolytic *“B. hampsonii”* strain 30599) on this day, 1.86 × 10^8^ strain 30599 genomic equivalents/g. In inoculated pigs the severity of bloody diarrhea ranged from that containing a few flecks of blood to diffusely hemorrhagic with copious blood and mucus as shown.

Post-inoculation fecal scores for INOC pigs are summarized in Figure 1. Eight of 11 INOC pigs developed MHD (score 4) (Figure 2), and one INOC developed mucoid diarrhea (#22, score 3). Of the two remaining INOC pigs, one (#19) remained healthy throughout the study, and one (#670) had fecal consistency typical of wet cement (score 1) on four post-inoculation days. To distinguish these from the nine diarrheic INOC pigs, they are referred to as “INOC (without MHD)”. No CTRL pig demonstrated mucoid or bloody diarrhea after sham inoculation, although one had fecal consistency typical of wet cement (score 1) on D6 and D9, and one had runny diarrhea (score 2) on D4 and wet cement consistency on D8, but was normal on all other days. Mucohemorrhagic diarrhea was significantly more frequent in INOC than in CTRL pigs (*P* = 0.002).

On D0, one INOC pig (#672) unexpectedly presented with runny diarrhea and fecal samples were submitted for routine aerobic and anaerobic culture to PDS. The sample was negative for *Salmonella* spp. but *E. coli*. was isolated. PCR results indicated that the *E.coli* isolate was positive for virulence factors AIDA-I and STb. Fecal consistency from this pig scored 0 or 1 until D8, then progressed to MHD by D9. The median number of days from first inoculation to the demonstration of mucoid or mucohemorrhagic diarrhea in INOC was 8, with the shortest and longest incubation periods at 7 and 11 days respectively. The onset of MHD was generally acute with affected pigs progressing from relatively normal feces (score 0 or 1) to MHD within 2.4 days (range 1–4) (Figure 1).

Average daily gain between D0 and D8 was numerically decreased in INOC (359 ± 143 g/day) compared to CTRL (413 ± 123 g/day), but did not differ significantly between groups.

Serum from blood collected on D0 and at termination was PRRSv PCR and ELISA negative. Intestinal tissue samples collected at termination tested negative for *Salmonella* sp., *Lawsonia intracellularis* and PCV2 by culture or PCR.

### *Brachyspira* shedding patterns

*Brachyspira* spp. isolates were recovered from feces from 10 of 11 INOC pigs between D0 and D4. This was likely associated with pass-through of the inoculation dose. Eight of 11 were culture positive for *“B. hampsonii”* strain 30599 solely, two of 11 for *B. intermedia*, one of 11 for *“B. hampsonii”* strain 30599 and *B. pulli* on different days (Figure 1). Between D5 and termination, *“B. hampsonii”* strain 30599 was consistently isolated in the feces and from the colon of all INOC pigs that developed MHD (Figure 1). *B. intermedia* was isolated from the feces and colon of the two INOC pigs without MHD. *B. intermedia* was also isolated sporadically from the feces of 5 of 6 and from terminal colon in 3 of 6 CTRL pigs. The presence of *B. intermedia* in feces was not associated with diarrhea. Inadvertently, an issue with the culture vessel on D9 resulted in negative cultures from all pigs. Culture positive isolates confirmed as *“B. hampsonii”* strain 30599 had 99-100% sequence identity to the inoculum based on sequencing of the *nox* gene. Any minor differences in sequence are most likely explained by PCR artefact since the Taq DNA polymerase used was not a proofreading enzyme.

Quantifiable levels of *“B. hampsonii”* strain 30599 were detected by qPCR beginning D4 in 11 of 11 INOC pigs (Figure 1). “*B. hampsonii”* strain 30559 was not detected in CTRL pigs. In INOC pigs without MHD, quantifiable levels were detected on a single day in each pig (D8 or D9). In INOC pigs with MHD, quantifiable levels of *“B. hampsonii”* strain 30599 were detected over multiple consecutive days preceding euthanasia. On average, quantifiable levels were detected for four days prior to the onset of MHD (range 2 to 6). The average concentration of *“B. hampsonii”* strain 30599 across all days in INOC pigs with MHD was 8.05 (log 10 genomic equivalents/gram of feces).

Between D1 and D4, *“B. hampsonii”* strain 30599 was isolated by culture in 15 fecal samples of INOC pigs with MHD. In these same samples, the organism was detected by PCR 13 times. Of these, 3 samples had quantifiable levels of DNA whereas the remaining 10 had detectable but non-quantifiable levels (i.e. beyond the lower limit of the linear portion of the standard curve). From D5 to termination, *“B. hampsonii”* strain 30599 was isolated by culture in 37 fecal samples of INOC pigs with MHD, whereas DNA was detected in the same samples 41 times, always at quantifiable levels. These data indicate that PCR and culture had similar overall detection frequencies.

### Pathological findings

**Figure 3:**
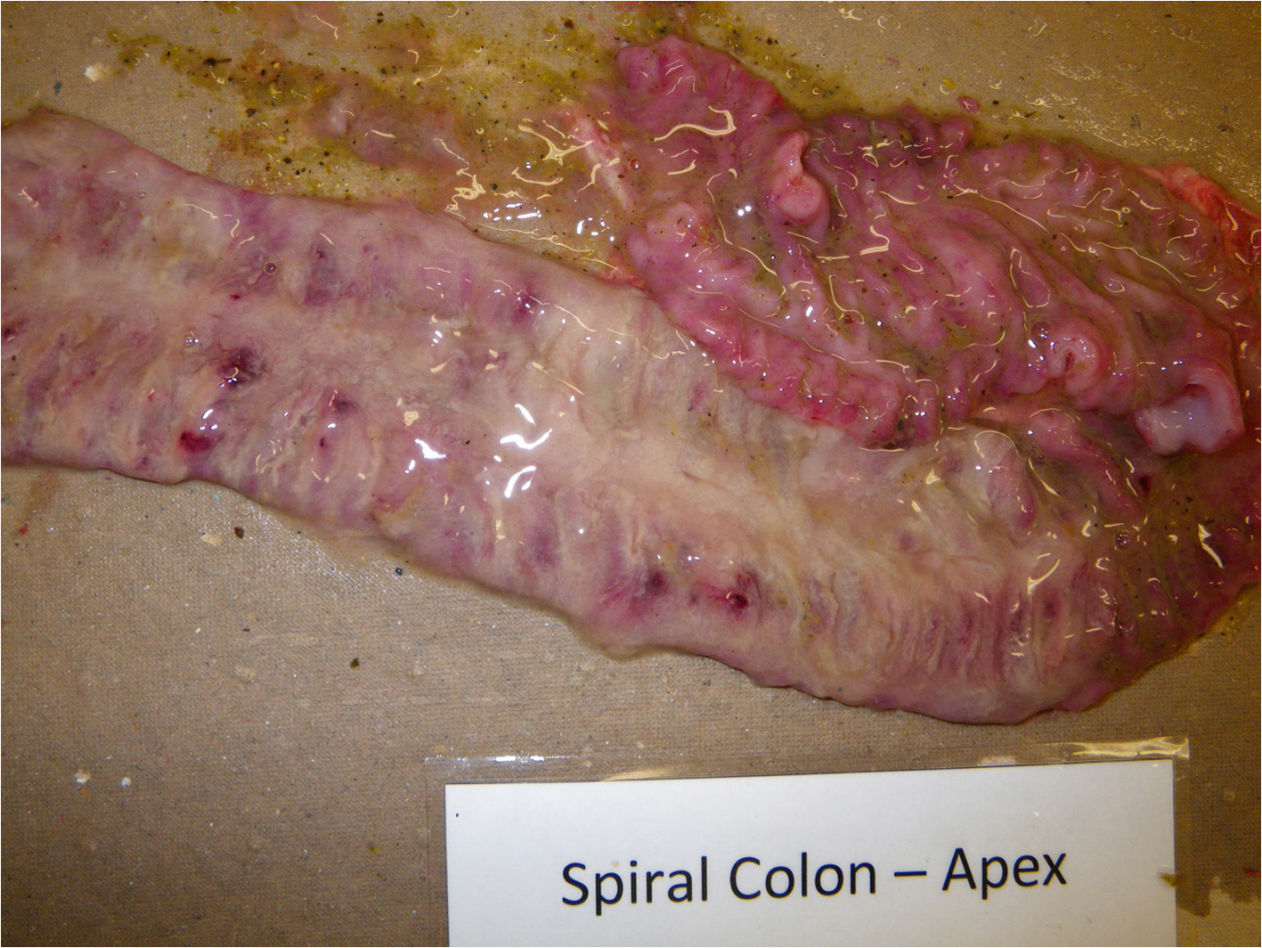
**Mucohemorrhagic colitis.** Congested mucosal surface of apex region of the spiral colon with thick layer of mucus and fibrin and occasional foci of haemorrhage. Pig #29, 12 days post inoculation, 48 hours post onset of mucohemorrhagic diarrhea.

A summary of gross pathological findings is presented in Table 1. Gross lesions in INOC pigs were confined to the large intestine, cecum and rectum. Cecum and rectum of INOC pigs displayed mild mucosal lesions, characterized as hyperemia associated with mucoid and fibrinous exudation. No lesions were observed in CTRL or in the small intestine of INOC pigs. In the two INOC without MHD, no lesions were observed in the proximal, apex or distal spiral colon; however, mild hyperemia of cecal mucosa was noted in one of these two pigs. Of the nine INOC with MHD, characteristic gross lesions of swine dysentery were observed consistently in spiral colon and cecum (Table 1). These lesions ranged in severity from mild, mucosal edema and hyperemia to severe, multifocal to coalescing, mucosal congestion with mucofibrinous exudate and focal areas of necrosis (Figure 3). In proximal, apex and distal spiral colon, gross lesions were significantly more frequent in INOC than in CTRL (*P* < 0.05 for all). In rectum, gross lesions were significantly more frequent in INOC with MHD than in CTRL (*P* = 0.04). Typhlitis was not a remarkable finding in INOC pigs, and lesion frequency did not differ between INOC and CTRL groups in this study. These results are contrary to previous descriptions of infections with *B. hyodysenteriae* or “*B. hampsonii”* clades I and II [2,10,11,22]. In INOC pigs with MHD, gross lesions were significantly more severe in the proximal and apex regions of the colon than in the distal colon (*P* < 0.01).

**Table 1:** Comparison of gross and histopathological lesion frequency in CTRL and INOC groups

		***P *** **value**				
**Description**		**CTRL (n = 6)**	**INOC (n = 11)**	**INOC (with MHD) (n = 9)**	**INOC vs. CTRL**	**INOC (with MHD) vs. CTRL**
Gross pathology frequency*						
	Proximal colon	1/6	8/11	8/9	0.04	0.01
	Apex colon	0/6	9/11	9/9	<0.01	<0.001
	Distal colon	0/6	7/11	7/9	0.02	<0.01
	Cecum	1/6	6/11	5/9	ns	ns
	Rectum	0/6	5/11	5/9	0.08	0.04
Histopathologic lesion frequency**						
	Colonic inflammation	0/6	9/11	9/9	<0.01	<0.001
	Colonic necrosis	0/6	9/11	9/9	<0.01	<0.001
	Cecal inflammation	0/6	6/11	6/9	0.04	0.001
	Cecal necrosis	0/6	3/11	3/9	ns	0.02
	Colonic spirochetes	0/6	8/11	8/9	<0.01	0.001

*Gross lesions include mucosal edema, hyperemia, congestion, with multifocal to diffuse mucofibrinonecrotic exudate.

**Histopathological lesions and colonic spirochete scores graded 0 to 3 reflecting negative, mild, moderate and severe inflammation or necrosis. Scores greater than 1 were considered lesional.

**Figure 4:**
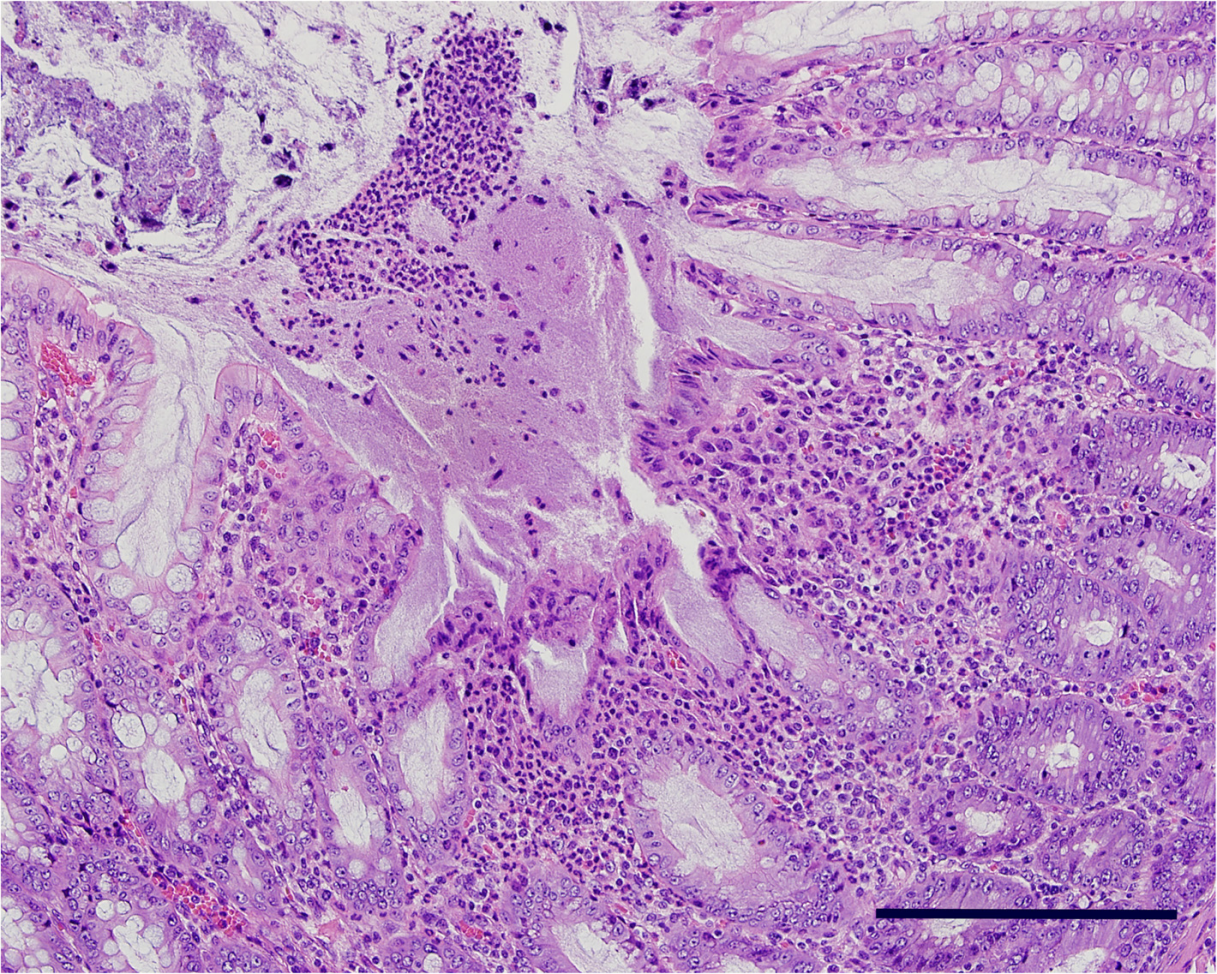
***Brachyspira *colitis.** In a section of proximal colon (Pig #671) there is a focal area of epithelial necrosis surrounded in the lamina propria by a mixed inflammatory infiltrate composed primarily of degenerate neutrophils, and covered by a thick mat of mucous containing degenerate neutrophils, necrotic epithelial cells, and bacterial colonies. Hematoxylin and Eosin. Bar = 200 μm.

**Figure 5:**
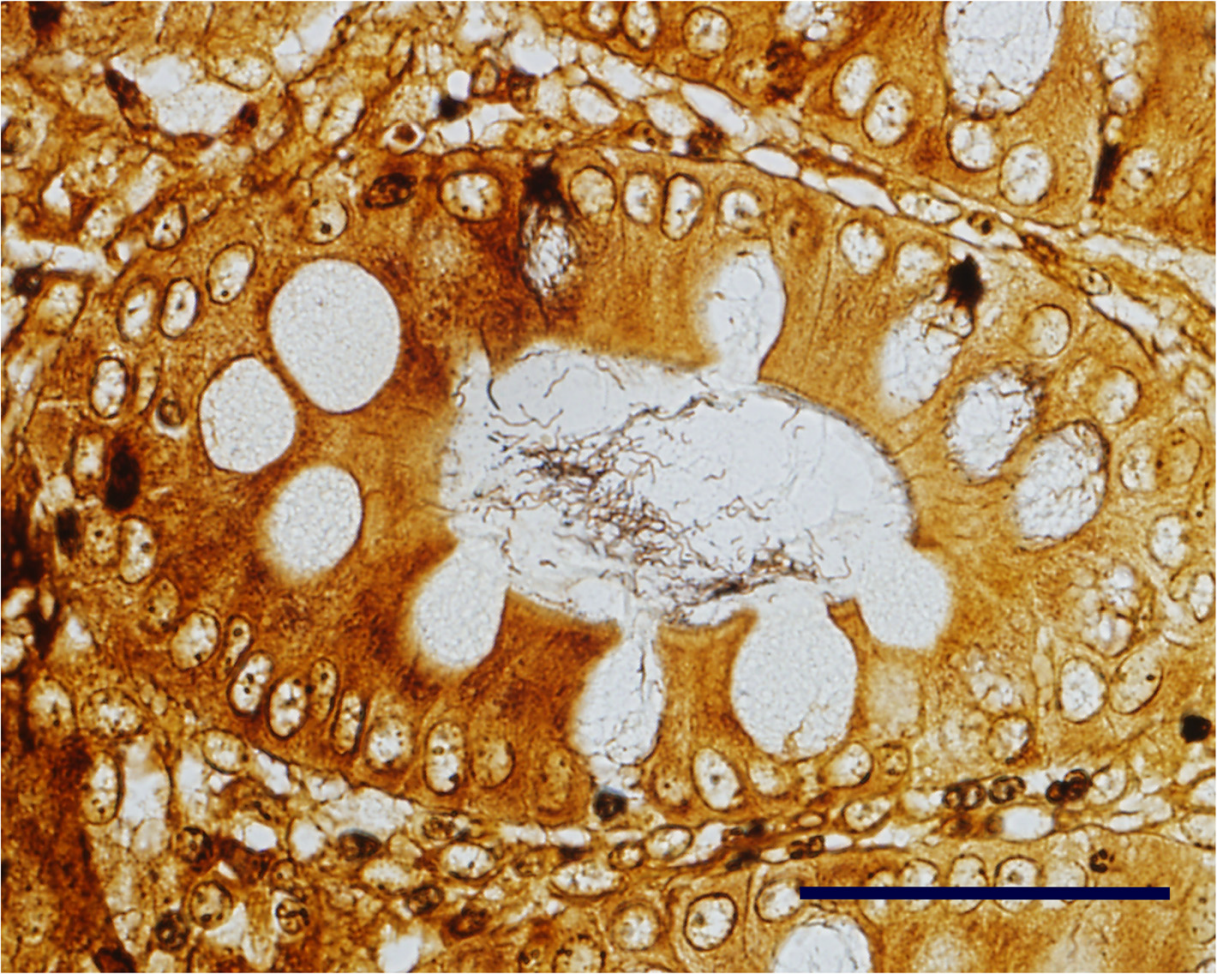
***Brachyspira *colitis.** In the same section of proximal colon as Figure 4, the tubular mucous-secreting glands contain large numbers of long, thin spiral-shaped bacteria. Warthin-Faulkner. Bar = 100 μm.

The frequency and severity of histopathologic findings were consistent with clinical signs and macroscopic lesions (Table 1). A thick layer of mucus and neutrophils (Figure 4) was observed on the colonic mucosa in all but one INOC pig. The nine of eleven pigs with positive cultures had histologic scores of 2 to 3 in the colon. By contrast, five of six CTRL pigs demonstrated mats of Gram-negative rod-shaped bacteria with minimal to no mucus (histologic score of 1). The presence of inflammation and superficial necrosis in the colon and cecum was higher in INOC than CTRL pigs (Table 1), as were numbers of spirochetes visualized in colon using silver staining (Table 1, Figure 5). Inflammation and necrosis were more severe in colon than cecum (*P* < 0.01).

### Evaluation of *ante mortem* sampling techniques

One-hundred and sixty-five samples (55 GenoTube swabs, 55 culture swabs, 55 fecal samples) were collected. Since there were no diarrheic pigs on the majority of sampling days, 78% of samples (43/55) were obtained from pigs with normal feces (score 0 or 1), whereas 9% (5/55) originated from pigs with loose to watery feces (score 2) and 13% (7/55) from pigs with mucoid or mucohemorrhagic diarrhea (score 3 or 4). Forty-two percent (23/55) of the fecal samples were collected prior to inoculation.

**Figure 6:**
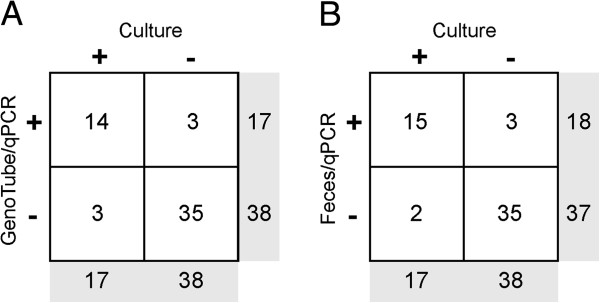
**Comparison of detection of “ *B. hampsonii *” strain 30599 by qPCR on DNA extracts from feces or GenoTube swabs, or culture on selective media.** Contingency tables show the numbers of samples determined to be positive or negative for *“B. hampsonii”* strain 30599 by **(A)** culture or qPCR on DNA extracts from GenoTube forensic swabs, and **(B)** culture or qPCR on DNA extracts from fecal samples.

Culture on BJ media followed by sequencing of the *nox* gene was set as the diagnostic gold standard for detection of *“B. hampsonii”* clade I strain 30599. A comparison of detection rate of *“B. hampsonii”* strain 30599 across the three *ante mortem* sampling methodologies is shown in Figure 6. Only 12 samples were collected from pigs with runny, mucoid or mucohemorrhagic diarrhea (score >1). Two of these were negative by culture, two were negative by GenoTube PCR, and one was negative by PCR of fecal DNA extracts. Both PCR methodologies detected *“B. hampsonii”* strain 30599 in all samples of mucoid and mucohemorrhagic diarrhea (score 3 or 4). On the other hand, one sample of mucohemorrhagic diarrhea collected on D8 was negative by culture, but positive by both PCR methodologies. This sample had 5.07 × 10^7^ genome equivalents/g of feces based on qPCR. While this dataset is small, biased towards negative test results, and limited to a single experiment, the results suggest these assays have similar detection capabilities.

## Discussion

The results of this experiment confirm that *“B. hampsonii”* clade I strain 30599 is pathogenic in susceptible pigs, and results in clinical disease indistinguishable from swine dysentery associated with *B. hyodysenteriae* and *“B. hampsonii”* clade II. This finding is relevant for pork producers in Canada and elsewhere whose herds experience mucohemorrhagic diarrhea. To our knowledge this is the first report of experimental reproduction of disease associated with Canadian strain 30599. Moreover, a previous report from Burrough *et al.*[11] pre-dated our present understanding based on *nox* sequences, that the strongly β-hemolyic strains of *B. intermedia* (KC35, EB106) used in these experiments were in fact *“B. hampsonii”* clade I. We hope the present research will help to clarify any potential confusion that may exist regarding the pathogenicity of this organism.

A second substantial deliverable of this research is the first description of a novel quantitative PCR assay specific for *“B. hampsonii”* clade I. Based on a discriminating region of the genome, this SYBR-based PCR assay enables quantification, and does not cross react with other known *Brachyspira* species including *“B. hampsonii”* clade II.

Three *Brachyspira* species are capable of causing bloody diarrhea: *B. hyodysenteriae, “B. hampsonii”* clades I and II, and *“B. suanatina”. B. hyodysenteriae* is globally distributed, while *“B. suanatina”* is reported only in Europe. Although *“B. hampsonii”* was discovered and mainly recognized in North America, isolations of clade I in Europe have been reported recently. One report details the isolation from two gilts that were imported from the Czech Republic to Belgium with soft watery feces within dilated large intestines [14]. The second account describes the isolation from a group of 17 grower pigs demonstrating mild to moderate diarrhea imported from Belgium to Germany [23]. A third study described the first finding of “*B. hampsonii”* from feces of birds, more specifically graylag geese and mallards in northwestern Spain [24].

In the present study, the onset of mucoid or mucohemorrhagic diarrhea in INOC pigs occurred within 7–11 days of inoculation, and was preceded by the fecal shedding of high levels (10^5^ to 10^8^ genomic equivalents/gram of feces) of *“B. hampsonii”* strain 30599 for up to 6 days. The prevalence of MHD was similar to that observed in the *“B. hampsonii”* clade II strain 30446 experimental reproduction [10] that used a similar animal model. It must be noted that this animal model does not require the feeding of 100% soybean meal prior to inoculation although a 16-hour period of fasting to enhance gastric motility is performed prior to each inoculation.

Swine dysentery is a multifactorial disease, with clinical expression dependent on individual host factors such as the gut microbiota [25], environmental conditions including diet composition [26,27], and presence of a virulent organism. Interestingly, two challenged pigs did not develop diarrhea and from both, *B. intermedia* was isolated in feces and in colonic tissue collected at termination. *B. intermedia* is generally accepted to be a gut commensal and non-pathogenic to pigs [28-30]. Whether or not it played a role in preventing the development of diarrhea in these pigs is not known, but *B. intermedia* was also isolated in five control pigs, suggesting it was a normal inhabitant of the pigs used in this study. A wide range of *Brachyspira* species can be isolated from healthy pigs from commercial farms [31] and are presumed to contribute to the commensal microbiota. The isolation of species other than that contained in the inoculum should therefore not be unexpected.

One pig (#22) developed a mucoid diarrhea with no evidence of blood prior to recovery over several days. This differed from pigs experimentally inoculated with *“B. hampsonii”* clade II, where 100% of the animals that displayed diarrhea progressed to blood-stained feces within 8 days of inoculation [10]. Field veterinarians, upon submission of diagnostic samples in western Canada, have often communicated variation in clinical presentation, particularly diarrhea associated with *“B. hampsonii”* clade I. We have further studies underway to help improve the understanding of the disease pathogenesis, and potential differences in pathogenic mechanisms associated with the pathogenic *Brachyspira* species.

Inoculated pigs with diarrhea had numerically lower ADG compared to non-challenged controls. However, due to study design limitations, experimental power was low and we could not investigate long-term effects of *“B. hampsonii”* clade I on the ADG of pigs. There are numerous reasons why ADG may be chronically depressed in surviving pigs, including any potential effects of *“B. hampsonii”* on the colonic mucosa and microbiota. These are relevant issues for the swine industry that are certainly worthy of future research.

The performance of qPCR on DNA extracted from GenoTube swabs was found to be comparable to qPCR on DNA extracted from feces, or culture. Rectal swabs are an easy and quick tool, enabling efficient sampling of large numbers of pigs, with minimal stress to the pigs and collectors. Moreover, a large percentage of laboratory errors occur during the pre-analytical phase of diagnosis, prior to samples arriving at the diagnostic laboratory [15,32]. This, and the fact that GenoTube swabs preserve DNA eliminating the need for refrigeration, makes it a practical, yet reliable methodology for sampling pigs with *Brachyspira*-associated diarrhea. However, the methodology does have several important limitations including the inability to isolate by culture. In addition, the use of rectal swabs precludes the ability to quantify target DNA in feces. Quantification may be important diagnostically since *“B. hampsonii”* clade I or II can be detected at low concentration (<10^5^ genomic equivalents/gram) in healthy, non-diarrheic weaner or finisher pigs, whereas levels greater than 10^5^ genomic equivalents/gram of feces or tissues [4].

## Conclusions

In summary, recent reports describing the emergence of *“Brachyspira hampsonii”* in North America and Europe provide evidence of the importance of this pathogen for the global pork industry. Results of this study confirm the pathogenicity of a Canadian *“B. hampsonii”* clade I strain 30599 and describe the course of disease following experimental challenge. We confirmed that strain 30599 caused mucohemorrhagic diarrhea indistinguishable from swine dysentery associated with *B. hyodysenteriae* or *“B. hampsonii”* clade II strain 30446. All pigs with bloody diarrhea were both qPCR and culture positive. Gross pathology and histopathology revealed characteristic lesions that were more severe in the proximal and apex regions of the colon, than the distal colon. Testing for other relevant pathogens, including PRRSv, *L. intracellularis*, PCV2 and *Salmonella* sp. were negative. Additionally, we evaluated several *ante mortem* sampling techniques and confirmed that a forensic swab, GenoTube Livestock, designed to preserve DNA during shipping may be a useful tool for diagnostic and surveillance projects, especially in settings where timely transport of diagnostic samples is challenging. Further evaluation of GenoTube swabs in field situations that do not require *Brachyspira* isolation and DNA quantification is warranted.

## Abbreviations

ADG: Average daily gain; Bp: Base pair; CTRL: Group serving as control; D: Day; H & E: Hematoxylin and Eosin stain; INOC: Treatment group inoculated with *“B. hampsonii”* clade I strain 30599; MHD: Mucohemorrhagic diarrhea; PCR: Polymerase chain reaction; PCV2: Porcine circovirus type 2; PDS: Prairie Diagnostic Services, Inc., Saskatoon, Canada; PRRSv: Porcine reproductive and respiratory syndrome virus; SD: Swine dysentery; WF: Warthin Faulkner silver stain.

## Competing interests

The University of Saskatchewan has intellectual property applications pertaining to the *“B. hampsonii”* clade II Canadian type strain (*Brachyspira* sp. sask30446) titled: “Diagnostic Method for Colitis” (PTC CA/2011/000828) submitted in July 2011 and “Isolated *Brachyspira* and methods and compositions for expanding and isolating *Brachyspira*” (PCT WO/2013/010260) submitted in July 2012. Both are published and available online at: http://patentscope.wipo.int. JCSH, JER and JEH are named co-inventors. This does not alter the authors’ adherence to all policies pertaining to publication, interpretation of results, or sharing of materials and reagents.

## Authors’ contributions

*In vivo* work was conducted by HDL, MOC and JCSH. “*Brachyspira hampsonii”* strain 305996 was initially isolated and propagated for the trial by JER. Laboratory work was conducted by CF, HDL and JEH. MOC evaluated the *ante mortem* sample methodologies. SED performed pathological assessments. JEH and JCSH are Principal Investigators. All authors read and approved the final manuscript.

## Supplementary Material

Additional file 1 Animal Research: Reporting In Vivo Experiments (ARRIVE) Checklist for present experiment. Click here for file
